# Impaired OMA1-dependent cleavage of OPA1 and reduced DRP1 fission activity combine to prevent mitophagy in cells that are dependent on oxidative phosphorylation

**DOI:** 10.1242/jcs.144337

**Published:** 2014-05-15

**Authors:** Thomas D. B. MacVicar, Jon D. Lane

**Affiliations:** Cell Biology Laboratories, University of Bristol, University Walk, Bristol BS8 1TD, UK

**Keywords:** Autophagy, Mitophagy, Mitochondrial dynamics, OPA1, OMA1, DRP1

## Abstract

Mitochondrial dynamics play crucial roles in mitophagy-based mitochondrial quality control, but how these pathways are regulated to meet cellular energy demands remains obscure. Using non-transformed human RPE1 cells, we report that upregulation of mitochondrial oxidative phosphorylation alters mitochondrial dynamics to inhibit Parkin-mediated mitophagy. Despite the basal mitophagy rates remaining stable upon the switch to dependence on oxidative phosphorylation, mitochondria resist fragmentation when RPE1 cells are treated with the protonophore carbonyl cyanide *m*-chlorophenyl hydrazone. Mechanistically, we show that this is because cleavage of the inner membrane fusion factor L-OPA1 is prevented due to the failure to activate the inner membrane protease OMA1 in mitochondria that have a collapsed membrane potential. In parallel, mitochondria that use oxidative phosphorylation are protected from damage-induced fission through the impaired recruitment and activation of mitochondrial DRP1. Using OMA1-deficient MEF cells, we show that the preservation of a stable pool of L-OPA1 at the inner mitochondrial membrane is sufficient to delay mitophagy, even in the presence of Parkin. The capacity of cells that are dependent on oxidative phosphorylation to maintain substantial mitochondrial content in the face of acute damage has important implications for mitochondrial quality control *in vivo*.

## INTRODUCTION

Mitochondria are highly dynamic organelles that generate ATP through oxidative phosphorylation (OXPHOS), contribute to lipid and Ca^2+^ homeostasis, and mediate and/or amplify cellular apoptotic responses (Youle and van der Bliek, 2012). It is essential that cells maintain a healthy population of mitochondria, and this is achieved through conserved repair pathways, and coordinated pathways of biosynthesis and degradation. The catabolic process of macroautophagy (‘autophagy’ hereafter) plays a key role in mitochondrial quality control, as it has the unique capability to isolate damaged and/or redundant mitochondria and deliver them to the lysosome for degradation through a selective pathway called ‘mitophagy’ (Lemasters, 2005; Youle and Narendra, 2011). Disturbances in the relationship between quality control and energy homeostasis contribute to disease (Gautier et al., 2008; Youle and Narendra, 2011), highlighting the importance of understanding how cellular energy demands and mitophagy are coupled.

Mitophagy is coordinated by an expanding family of adaptor molecules that flag damaged mitochondria for recognition by the autophagy machinery through conserved LC3-interacting regions (LIRs) (Johansen and Lamark, 2011; Youle and Narendra, 2011). The adaptors include Nix (BNIP3L) (Novak et al., 2010; Sandoval et al., 2008; Schweers et al., 2007) and FUNDC1 (Liu et al., 2012) in mammalian cells, and Atg32 in yeast (Aoki et al., 2011; Kanki et al., 2009; Okamoto et al., 2009). In a highly efficient and well-characterised mitophagy pathway, the serine/threonine kinase PINK1 interrogates mitochondria for bioenergetic functionality through the highly selective recruitment of the ubiquitin E3 ligase Parkin (PARK2) to the mitochondrial surface (Narendra et al., 2008). Mechanistically, healthy mitochondria constitutively import PINK1 into their inner membrane (IMM), where cleavage by PARL proteases (Jin et al., 2010) triggers retro-translocation into the cytoplasm and proteasomal degradation of PINK1 (Yamano and Youle, 2013), but those with a collapsed membrane potential (Δψ_m_) accumulate PINK1 on the outer membrane (OMM), from where it recruits Parkin, triggering substrate ubiquitylation and enrichment of the LIR-containing adaptor, p62/SQSTM1 (Jin et al., 2010; Youle and Narendra, 2011). The efficiency of this pathway becomes clear when mammalian cells that overexpress Parkin are treated with uncoupling reagents, such as the protonophore carbonyl cyanide *m*-chlorophenyl hydrazone (CCCP), which triggers degradation of the entire mitochondrial content within 24 hours (Narendra et al., 2008).

In most cells, the mitochondrial network is continually remodelled through regulated pathways of fusion, fission and transport. At the molecular level, fusion and fission are controlled by dynamin family GTPases, with DRP1 and mitofusins (Mfn1 and Mfn2) controlling fission and fusion of the OMM, respectively, and OPA1 driving fusion of the IMM (Youle and van der Bliek, 2012). Diverse regulatory pathways influence mitochondrial dynamics through these proteins, with phosphorylation, sumoylation and proteasome-dependent and independent degradation playing prominent roles (Youle and van der Bliek, 2012). Importantly, pathways that link mitochondrial dynamics with quality control are beginning to emerge (Gomes and Scorrano, 2013). For example, mitochondrial fission has been shown to be necessary, but not sufficient, for mitophagy (Frank et al., 2012; Gomes et al., 2011; Narendra et al., 2008; Rambold et al., 2011; Twig et al., 2008). Indeed, the relationship between fission and mitophagy is, perhaps, best demonstrated during cell stress when fusion–fission dynamics are altered to generate a hyperfused mitochondrial network that potently restricts mitophagy (Gomes et al., 2011; Jahani-Asl et al., 2007; Rambold et al., 2011; Tondera et al., 2009). This has led to the idea that control of the size of mitochondria is a trivial, but, nevertheless, fundamental feature of the mitophagy response.

Increasing cellular demand for mitochondrial OXPHOS triggers changes in mitochondrial ultrastructure and network dynamics to generate a more efficient mitochondrial population (Cogliati et al., 2013; Hackenbrock et al., 1971; Rossignol et al., 2004). Given the links between mitochondrial dynamics and quality control, it is likely that mitophagy is coordinated differently with respect to OXPHOS activity in cells. Indeed, in yeast, mitophagy is actively suppressed during OXPHOS-dependent growth (Kanki and Klionsky, 2008; Kanki et al., 2009; Okamoto et al., 2009); however, the underlying mechanisms of, and the relationships between OXPHOS activity and mitophagy in mammalian cells remain obscure. Crucially, and despite mitochondrial OXPHOS being the most efficient pathway for energy generation, many cultured cell lines – particularly those derived from human cancers – rely on aerobic glycolysis for ATP supply (Bensinger and Christofk, 2012). Consequently, studies exploring the regulation of mitochondrial dynamics and mitophagy in cell culture are typically carried out in a background of upregulated aerobic glycolysis and suppressed mitochondrial OXPHOS. Shifting cellular energy dependency towards OXPHOS can be achieved simply by adapting cells to grow on galactose, instead of the widely used monosaccharide glucose. In galactose media, cells suppress glycolysis and engage glutaminolysis to drive the citric acid cycle (Melser et al., 2013; Reitzer et al., 1979). Using this approach, Melser and colleagues have recently reported that HeLa cells upregulate basal mitophagy to improve the efficiency of ATP synthesis when transferred to OXPHOS-dependent culture conditions (Melser et al., 2013). This correlated with a dramatic stimulation of basal autophagy and required Nix/BNIP3L, as well as recruitment of the small GTPase Rheb to the OMM (Melser et al., 2013). Here, we show that shifting the energy dependency from aerobic glycolysis towards mitochondrial OXPHOS impairs stress-induced mitophagy. In OXPHOS-dependent cells, mitochondrial dynamics are biased against fission following damage, thereby protecting a population of OXPHOS-active mitochondria from mitophagy. This is controlled through the suppression of DRP1 activity and the protection of fusion-competent, long-form OPA1 at the IMM. Notably, our data point to a key role for the processing of OPA1 in the coordination of mitochondrial dynamics and mitophagy in stressed cells. These findings will have important implications for our understanding of the relationships between mitochondrial OXPHOS activity, mitochondrial network dynamics and mitochondrial quality control.

## RESULTS

### Parkin-mediated mitophagy is suppressed in OXPHOS-dependent RPE1 cells

**Fig. 1.**
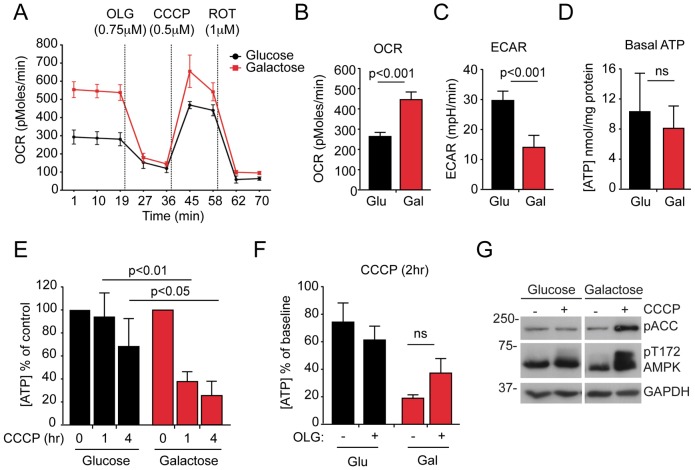
**Bioenergetic parameters of glucose- and galactose-cultured RPE1 cells.** (A) Oxygen consumption rate (OCR) trace for glucose- and galactose-cultured (Glu and Gal, respectively) YFP–Parkin RPE1 cells, measured using a Seahorse Bioscience XF24 Extracellular Flux Analyzer. The injection order of oligomycin (OLG), CCCP and rotenone (ROT), and the optimised concentrations for each toxin are indicated (*n* = 4 wells). (B) The basal OCR in four wells of glucose- and galactose-cultured YFP–Parkin RPE1 cells (means of three timepoints±s.d.; *P* values were calculated using Student's *t*-test). (C) The basal extracellular acidification rate (ECAR) was measured simultaneously to the OCR in the same wells of glucose- and galactose-cultured cells (means of three timepoints±s.d., *P* values were calculated using Student's *t*-test). (D) Similar steady state ATP levels in glucose- and galactose-cultured RPE1 cells were measured. Means of three timepoints±s.d.; ns, not significant. (E) RPE1 cells that had been grown in galactose exhibited a significantly greater drop in cellular [ATP] after treatment with CCCP compared with glucose-fed cells (*n* = 4; means±s.e.m.; two-way ANOVA, Bonferroni post-test). (F) ATP concentrations in glucose- and galactose-fed RPE1 cells after treatment with CCCP alone or CCCP with 10 µM OLG shown as the percentage of DMSO-treated cells. (G) Evidence for increased AMPK activation. Phosphorylation at residue Thr172 of AMPK and of the AMPK substrate acetyl-CoA carboxylase (pACC) in OXPHOS-dependent RPE1 cells that had been treated with CCCP.

To explore the relationships between mitochondrial network dynamics and quality control, we measured mitophagy in several human cell lines that stably expressed yellow fluorescent protein (YFP)-tagged Parkin and found human telomerase immortalised retinal pigment epithelial (hTERT-RPE1, herein referred to as RPE1) cells to be the most efficient (80–90% cells depleted all mitochondria within 24 hours of treatment with CCCP; supplementary material Fig. S1). Blocking autophagic flux through the lysosomal system by inhibiting the lysosomal H^+^-ATPase using Bafilomycin A1 (BafA1) (supplementary material Fig. S2A), or reducing the efficiency of autophagosome assembly by using siRNA-mediated suppression of the core autophagy gene *ATG7* (supplementary material Fig. S2B) reduced the mitophagy response, as expected. To test how cellular bioenergetics influence mitophagy, we cultured YFP–Parkin-expressing RPE1 cells in medium containing 10 mM glucose or 10 mM galactose, in the presence of equal amounts of glutamine (therefore, the only difference was the type of monosaccharide sugar). Bioenergetic profiling demonstrated that RPE1 cells that were grown on glucose mainly utilised aerobic glycolysis, as they exhibited a low oligomycin-sensitive oxygen consumption rate and a relatively high extracellular acidification rate (ECAR; Fig. 1A–C). Upon conditioning to galactose medium, the same cells shifted their energy dependency towards mitochondrial OXPHOS, with increased oxygen consumption, a drop in the ECAR, and a greater maximal respiration rate and capacity (Fig. 1A–C). Little variation in basal [ATP] was recorded between these conditions (Fig. 1D), but galactose-conditioned cells showed a sharper, partially oligomycin-protected decline in [ATP] during CCCP treatment (Fig. 1E,F), correlating with evidence of increased AMPK activity (Fig. 1G).

The observation that HeLa cells dramatically upregulate basal mitophagy upon shift to galactose medium (Melser et al., 2013) prompted us to first check whether steady state mitochondrial turnover was also altered in OXPHOS-dependent RPE1 cells. Immunoblotting for various mitochondrial proteins demonstrated that the activation of OXPHOS did not alter steady state mitochondrial turnover in RPE1 cells (supplementary material Fig. S3A). The expression of the mitochondrial markers HSP60, Tom20 and cyclophilin D (CypD) was reduced during cycloheximide treatment in both conditions, but in a manner that was independent of the mitophagy–lysosomal system, because the levels were not restored by treatment with BafA1 (supplementary material Fig. S3A) [the apparent restoration of the levels of the mitochondrial phosphate–carrier protein PiC (also known as SLC25A3) in galactose-containing medium was not statistically significant]. Mitochondrial–lysosomal colocalisation analysis also suggested that basal mitophagy was not altered by the switch to galactose medium (supplementary material Fig. S3B), which argues against the hypothesis that there is an upregulation of mitophagy upon the shift to OXPHOS conditions in wild-type RPE1 cells.

**Fig. 2.**
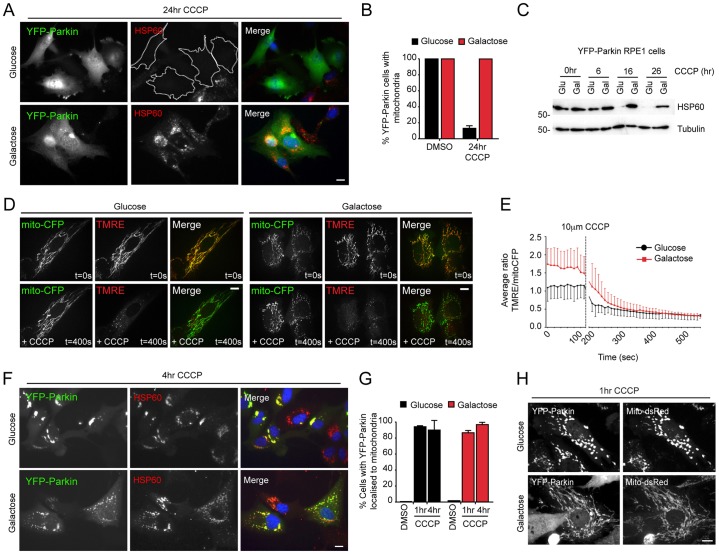
**Parkin-mediated mitophagy is inhibited in OXPHOS-dependent RPE1 cells.** (A,B) Analysis of mitophagy by using wide-field fluorescence microscopy of glucose- and galactose-cultured YFP–Parkin-expressing RPE1 cells that had been treated with 10 µM CCCP (A, example fields; B, quantification; *n* = 3; means±s.d.). (C) Analysis of mitophagy by immunoblotting for HSP60 in glucose- and galactose-cultured YFP–Parkin-expressing RPE1 cells that had been treated with 10 µM CCCP. (D,E) Δψ_m_ dissipation rates in glucose- and galactose-cultured RPE1 cells. (D) Example stills from live-cell movies of glucose- and galactose-cultured RPE1 cells expressing YFP–Parkin and mito–CFP that had been loaded with 50 ng/ml TMRE and treated with 10 µM CCCP (YFP–Parkin not shown). The movies were obtained with a spinning disc confocal microscope. (E) The ratio of TMRE∶mito–CFP was calculated for the mitochondria in each movie, and the means of each timepoint±s.d. are shown (8–9 cells across two experiments). Similar rates of loss of TMRE fluorescence were recorded in cells that had been grown on both energy substrates. (F,G) YFP–Parkin recruitment kinetics were assessed by fluorescence imaging (F, example fields; G, quantification; *n* = 3; means±s.d.). Note the different distribution of mitochondria that are decorated with YFP–Parkin between conditions (see text). (H) YFP–Parkin-decorated mitochondria remain elongated in OXPHOS-dependent cells at early timepoints after treatment with CCCP. RPE1 cells that stably expressed YFP–Parkin and mito–CFP were imaged by using confocal microscopy. Scale bars: 10 µm.

Using YFP–Parkin RPE1 cells that had been cultured in either glucose- or galactose-based media, we next asked how OXPHOS dependency influences mitophagy during CCCP-induced mitochondrial stress. Strikingly, and in stark contrast with their glucose-cultured equivalents, all of the YFP–Parkin-expressing RPE1 cells that had been grown on galactose retained a significant population of mitochondria following 24 hours of treatment with CCCP (Fig. 2A–C). This acute block in Parkin-mediated mitophagy occurred despite comparable rates of Δψ_m_ dissipation (Fig. 2D,E). Crucially, we observed similar rates of mitochondrial YFP–Parkin recruitment in RPE1 cells that had been treated with CCCP and grown on either glucose or galactose (Fig. 2F,G). This differs from a previous report that documented a lack of mitochondrial Parkin recruitment in galactose-cultured HeLa cells that had been treated with CCCP (Van Laar et al., 2011), suggesting cell-type variability in the PINK1–Parkin pathway, with respect to mitochondrial function. Interestingly, the structure and distribution of Parkin-decorated mitochondria clearly differed at the early timepoints after treatment with CCCP between growth conditions – in glucose, rapid mitochondrial fragmentation and perinuclear clustering was observed (as previously reported by Narendra et al., 2008), whereas, in galactose, Parkin-decorated mitochondria remained extended and reticular, and failed to cluster in the perinuclear region (Fig. 2F,H).

### A functional autophagy pathway in OXPHOS-dependent RPE1 cells

One possible explanation for the suppression of Parkin-mediated mitophagy under condition of OXPHOS was a block in autophagy. To test this, we immunoblotted RPE1 cell lysates for lipidated LC3 (LC3-II) (supplementary material Fig. S4A,B), and immunostained RPE1 cells for LC3 and the early autophagosome marker WIPI2 (Polson et al., 2010) (supplementary material Fig. S4C,D). Cells that had been cultured in glucose or galactose demonstrated similar autophagic responses to treatment with CCCP and to amino acid starvation, as quantified by the generation of LC3-II (supplementary material Fig. S4A,B) and the formation of LC3–WIPI2 puncta (supplementary material Fig. S4C,D). Importantly, this approach, combined with the use of an RPE1 cell line that stably expressed mCherry–GFP–LC3, demonstrated that there was no difference in the autophagic flux between glucose- and galactose-fed RPE1 cells (supplementary material Fig. S4D,E). Importantly, both p62 and LC3 were recruited to mitochondria that were decorated with YFP–Parkin at early timepoints upon treatment with CCCP (1 hour) in galactose-cultured RPE1 cells (supplementary material Fig. S4F), demonstrating that the early mitophagy signalling pathway is likely to be intact. Despite this, in galactose-cultured RPE1 cells that expressed cyan fluorescent protein (CFP)-tagged Parkin, we recorded a reduced colocalisation between mitochondrial Tom20 and mCherry–LC3-labelled autophagolysosomes after 3 hours of treatment with CCCP (supplementary material Fig. S4G). We, therefore, concluded that the inhibition of Parkin-mediated mitophagy in OXPHOS-active RPE1 cells was not due to a general block in autophagy, but that the incorporation of damaged mitochondria into nascent autophagosomes was somehow impaired.

### Impaired processing of L-OPA1 correlates with reduced mitochondrial fragmentation in OXPHOS-active cells

**Fig. 3.**
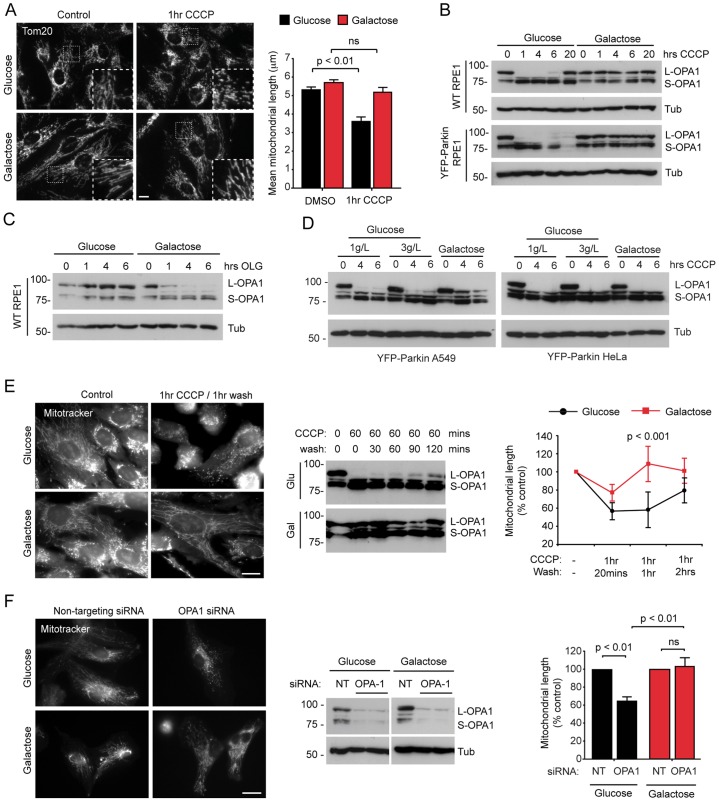
**Impaired L-OPA1 processing is linked to sustained mitochondrial length in OXPHOS-active RPE1 cells.** (A) Analysis of CCCP-induced mitochondrial fragmentation in glucose- and galactose-cultured wild-type RPE1 cells that had been stained for Tom20. Example images are shown in the right panel and the insets show enlarged images of the boxed areas. Quantification of the mitochondrial length is shown in the left panel. (means±s.d.; *n* = 3, ≥120 cells; *P* values calculated by using two-way ANOVA, Bonferroni post-test; ns, not significant.) (B) CCCP-induced L-OPA1 processing in glucose- and galactose-cultured wild-type (WT) and YFP–Parkin-expressing RPE1 cells. Cell lysates were immunoblotted with the antibodies shown. Tub, tubulin. (C) L-OPA1 processing in glucose- and galactose-cultured wild-type RPE1 cells that had been treated with oligomycin (OLG). (D) L-OPA1 processing in glucose- and galactose-cultured YFP–Parkin-expressing A549 (left panel) and HeLa (right panel) cells that had been treated with CCCP. Cells were grown with either 1 g/l or 3 g/l of glucose. (E) Recovery of mitochondrial length following CCCP withdrawal (1hr CCCP/1hr wash) in glucose- and galactose-cultured wild-type RPE1 cells. Example images are shown in the left panel, immunoblotting of lysates is shown in the middle panel, quantification of mitochondrial length is shown in the panel on the right (means±s.d.; *n* = 3; ≥120 cells; two-way ANOVA, Bonferroni post-test). (F) siRNA against OPA1 does not induce mitochondrial fragmentation in OXPHOS-dependent cells. Example images are shown in the left panel, immunoblotting of lysates for OPA1 is shown in the middle panel and quantification of mitochondrial length is shown in the panel on the right as a percentage of control (*n* = 4; ≥100 cells; means±s.d.; one-way ANOVA, Tukey's post-test). ns, not significant; NT, non-targeting; Tub, tubulin. Scale bars: 10 µm (A,E,F).

Several recent studies have demonstrated that mitochondrial fission is necessary, but not sufficient, for mitophagy (Frank et al., 2012; Gomes et al., 2011; Narendra et al., 2008; Rambold et al., 2011; Twig et al., 2008). Importantly, diverse cellular stresses, including starvation, are known to alter mitochondrial dynamics to generate a hyperfused mitochondrial network that potently restricts mitophagy (Gomes et al., 2011; Rambold et al., 2011; Tondera et al., 2009). Given that we observed differences in mitochondrial structure and distribution after treatment with CCCP in our initial mitophagy assays (Fig. 2F,H), we used imaging analysis to compare the length of mitochondria at steady state, and at early timepoints following treatment with CCCP, in glycolytic and OXPHOS-dependent RPE1 cells. We found that the mean steady state length of mitochondria was very similar under the two growth conditions (Fig. 3A); however, mitochondrial fragmentation was significantly reduced in galactose-cultured RPE1 cells that had been treated with CCCP (Fig. 3A). Thus, although OXPHOS-dependent RPE1 cells did not exhibit a hyperfused mitochondrial network in basal conditions, these cells were able to maintain an elongated network after acute mitochondrial stress. To investigate this further, we focused on the intricate regulation of the fusion and fission machinery.

Following depolarisation, which is induced by reagents such as CCCP, mitochondrial fragmentation is driven by DRP1 (Ishihara et al., 2003) in a process that follows the inducible cleavage of OPA1 at the IMM (Ishihara et al., 2006). Mammalian cells express eight OPA1 splice forms (Delettre et al., 2001), each of which has either one or two cleavage sites (S1 and S2) (McBride and Soubannier, 2010). A proportion of the OPA1 isoforms is constitutively cleaved at S2 by the YME1L protease, generating fusion-competent mixed populations of long and short OPA1 (L-OPA1 and S-OPA1, respectively) (Griparic et al., 2007; Song et al., 2007). In respiratory-compromised mitochondria, L-OPA1 is cleaved at S1 by the inducible protease OMA1, which renders it incapable of supporting membrane fusion (Ehses et al., 2009; Head et al., 2009; Ishihara et al., 2006; Quirós et al., 2012). In our hands, and in common with previous reports, L-OPA1 was rapidly cleaved in glycolytic RPE1 cells that had been treated with CCCP (within 1 hour; Fig. 3B). By contrast, cleavage was not observed in OXPHOS-dependent RPE1 cells under the same conditions (Fig. 3B), suggesting that the failure to process L-OPA1 at the S1 site might contribute to impaired mitochondrial fragmentation (Fig. 3A) and defective mitophagy (Fig. 2A–C). Interestingly, oligomycin triggered efficient cleavage of L-OPA1 (within 4 hours) in OXPHOS-active, but not in glycolytic, RPE1 cells (Fig. 3C), suggesting that the L-OPA1 cleavage machinery is capable of responding to changes in mitochondrial ATP levels. Hence, upregulating OXPHOS in RPE1 cells changed the control of mitochondrial dynamics at the level of L-OPA1 cleavage in a manner that did not reflect an altered cellular [ATP] (Fig. 1E). Intriguingly, the influence of growth conditions on CCCP-induced L-OPA1 cleavage differed between cell lines – cleavage of L-OPA1 was absent in OXPHOS-dependent A549 cells but was equally efficient in HeLa cells that had been grown on glucose or galactose (Fig. 3D).

The functional significance of impaired L-OPA1 cleavage in OXPHOS-dependent RPE1 cells became clear in CCCP washout experiments – in OXPHOS-dependent cells, the recovery of mitochondrial length was significantly faster (from a higher base level) than in glycolytic cells, for which the recovery correlated with *de novo* L-OPA1 synthesis (Fig. 3E). Hence, the failure to process L-OPA1 during mitochondrial stress might be a pivotal control point for the prevention of mitophagy in cells that are reliant on mitochondria for ATP generation. Interestingly, although siRNA suppression of OPA1 expression caused a significant decrease in the length of mitochondria in glycolytic RPE1 cells, this was not the case for OXPHOS-dependent RPE1 cells (Fig. 3F). This suggested that other factors controlling mitochondrial dynamics might contribute to the differing control of mitochondrial network composition in this context (discussed below).

### DRP1 fission activity is suppressed in OXPHOS-dependent cells

To explain the resistance to mitochondrial fragmentation in OXPHOS-dependent RPE1 cells silenced for OPA1, we first looked at the stability of the mitofusin proteins in RPE1 cells that expressed YFP–Parkin. Mfn1 and Mfn2 are subject to proteasome-mediated degradation in a Parkin-dependent fashion in cells with dissipated Δψ_m_ (Tanaka et al., 2010). Importantly, we observed efficient proteasomal degradation of Mfn1 and Mfn2 in both glucose and galactose growth conditions in CCCP-treated RPE1 cells that expressed YFP–Parkin (supplementary material Fig. S5A,B), consistent with the comparable rates of Δψ_m_ dissipation (Fig. 2D) and Parkin recruitment (Fig. 2F,G). Upon closer inspection, we recorded a significant delay in Mfn1 degradation at early time-points (1 hour; supplementary material Fig. S5C); however, this was unlikely to account for the differences in mitochondrial fragmentation rates in experiments that employed wild-type RPE1 cells (Fig. 3A,E,F).

**Fig. 4.**
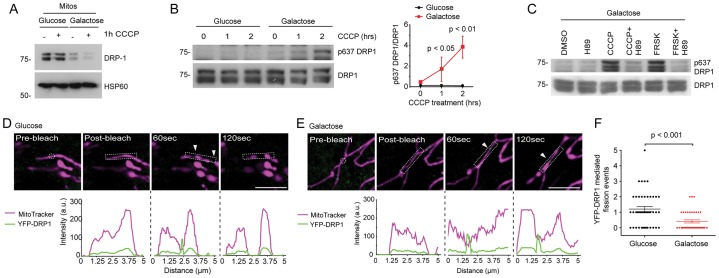
**DRP1 activity is suppressed in OXPHOS-active RPE1 cells.** (A) Reduced levels of mitochondrial DRP1 in OXPHOS-active RPE1 cells, before and after 1 hour of treatment with CCCP. Mitochondria that had been isolated from glucose- and galactose-cultured wild-type RPE1 cells were immunoblotted with the antibodies shown. (B) Phosphorylation of DRP1 on residue Ser637 in OXPHOS-active cells that had been treated with CCCP. An example immunoblot of cell lysates is shown on the left and densitometry quantification, normalised to the levels of total DRP1, is shown on the right (means±s.d., *n* = 3, two-way ANOVA, Bonferroni post-test). (C) Phosphorylation of DRP1 at residue Ser637 in CCCP-treated OXPHOS-active cells is PKA dependent. Galactose-cultured RPE1 cells were treated with the reagents shown, and the lysates were immunoblotted for total and phosphorylated DRP1 at Ser637 (H89, PKA inhibitor; FRSK, forskolin, PKA activator). (D,E) Laser photodamage and analysis of mitochondria fragmentation in RPE1 cells that stably expressed YFP–DRP1. Example spinning disc confocal images (top row) and line-scans (bottom row) are shown for glucose- (D) and galactose- (E) cultured cells. The times indicate the time post-bleach. a.u., arbitrary units. (F) Quantification of YFP–DRP1 fission events following laser photodamage in glucose- and galactose-cultured RPE1 cells. (Means±s.d., *n* = 3, ≥40 cells, *t*-test). Arrowheads indicate newly formed YFP punctae. Scale bars: 5 µm.

Inhibition of the mitochondrial fission factor DRP1 potently blocks mitophagy in cells that overexpress Parkin (Tanaka et al., 2010). We, therefore, tested whether DRP1-mediated fission kinetics differed between glycolytic and OXPHOS-active RPE1 cells. Notably, growth on galactose markedly reduced the recoverable pool of mitochondrial DRP1 (Fig. 4A), without affecting the total levels of DRP1 (data not shown). A similar scenario has been described for nitrogen-starved cells with hyperfused mitochondria (Rambold et al., 2011); however, this was attributed to elevated phosphorylation of residue Ser637 (Rambold et al., 2011), but the same was not true for OXPHOS-dependent RPE1 cells under basal conditions (Fig. 4B). Upon the addition of CCCP, we observed elevated PKA-dependent phosphorylation of residue Ser637, but only in the OXPHOS-active population (Fig. 4B,C), and this correlated with a further reduction in the levels of mitochondrial DRP1 (Fig. 4A). The phosphorylation event that occurred in response to treatment with CCCP for 2 hours in OXPHOS-active cells was dependent on PKA because it was abolished by 10 µM of the PKA inhibitor H89 (Fig. 4C). The sensitivity of DRP1 to PKA activity was also highlighted by the phosphorylation of residue Ser637 after 2 hours of treatment with 20 µM forskolin, an activator of PKA. To monitor mitochondrial DRP1 fission activity directly, we generated RPE1 cells that stably expressed YFP–DRP1 and subjected regions of their mitochondria to laser photodamage (Fig. 4D,E; supplementary material Movie 1 and 2). Although similar numbers of new mitochondrial YFP–DRP1 foci were scored in the vicinity of laser damage at steady state (data not shown), the progression of these into measurable fission events was significantly less likely in OXPHOS-active cells (Fig. 4D–F). Hence, a further functional consequence of the increased demand for OXPHOS is a reduction in DRP1 fission capability *in situ* following acute mitochondrial damage.

### Stabilisation of L-OPA1 in OXPHOS-active cells is controlled at the level of OMA1

**Fig. 5.**
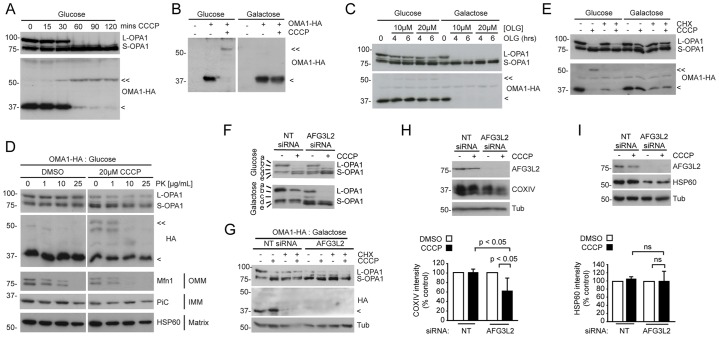
**Failure to activate 40-kDa OMA1 at the IMM explains the absence of L-OPA1 cleavage in OXPHOS-dependent RPE1 cells.** (A) Simultaneous L-OPA1 cleavage, 40-kDa OMA1 degradation and the generation of 60-kDa OMA1 in glycolytic RPE1 cells that stably expressed OMA1–HA and had been treated with CCCP. Lysates were immunoblotted with the indicated antibodies against OPA1 HA. (B) Impaired 40-kDa OMA1 degradation in OXPHOS-active RPE1 cells that stably expressed OMA1–HA (1 hour CCCP). (C) L-OPA1 is processed in the absence of detectable 60-kDa OMA1 in OXPHOS-active RPE1 cells that had been treated with oligomycin (OLG), as shown by immunoblotting. Glucose- and galactose-cultured RPE1 cells were incubated with 10 or 20 µM OLG for the times shown. (D) Protease protection assay. Mitochondria that had been isolated from control (DMSO) or CCCP-treated glycolytic RPE1 cells that stably expressed OMA1–HA were subjected to proteinase K (PK) treatment for the times indicated and then immunoblotted with antibodies against the indicated OMM, IMM and matrix resident proteins. (E) L-OPA1 processing is observed in the absence of detectable 60-kDa OMA1 in glycolytic RPE1 cells that had been treated with CCCP and cycloheximide (CHX) for 6 hours. (F–I) Silencing of AFG3L2 in OXPHOS-dependent RPE1 cells reveals a possible role for L-OPA1 cleavage in mitophagy. (F) Suppression of AFG3L2 expression enables L-OPA1 cleavage in CCCP-treated OXPHOS-dependent RPE1 cells. Immunoblotting for OPA1 reveals complex isoforms patterning. In galactose medium, band a (L-OPA1) is marginally decreased following treatment with AFG3L2 siRNA (note the appearance of band c), but is completely lost in the presence of CCCP (1 hour). (G) AFG3L2 siRNA triggers 40-kDa OMA1 degradation in OXPHOS-dependent RPE1 cells that stably expressed OMA1–HA. (H,I) Immunoblotting (top row) and densitometry-based quantification as a percentage of the untreated non-targeting siRNA control cells (bottom row) of (H) COXIV and (I) HSP60 levels as a measure of CCCP-induced mitophagy in AFG3L2-suppressed cells (20 hours) (means±s.d.; *n* = 3; one-way ANOVA, Tukey's post-test). <<, 60-kDa OMA1; <, 40-kDa OMA1; ns, not significant; NT, non-targeting; Tub, tubulin.

To understand how cellular energy status influences L-OPA1 processing, we examined the regulation of the inducible OPA1 protease OMA1. Synthesised as a 62-kDa precursor, OMA1 is efficiently processed following mitochondrial import to a ∼40-kDa transmembrane form that assembles multimeric complexes at the IMM (Kaser et al., 2003). Upon treatment with CCCP, ∼40-kDa OMA1 is rapidly degraded, and a ∼60-kDa species accumulates – events that are temporally linked to L-OPA1 cleavage (Head et al., 2009). In glycolytic RPE1 cells that stably expressed OMA1 tagged with hemagglutinin (HA), the loss of ∼40-kDa OMA1, the appearance of ∼60-kDa OMA1 and the inducible cleavage of L-OPA1 all took place within 30–60 minutes of CCCP addition (Fig. 5A). Strikingly, in OXPHOS-dependent RPE1 cells that stably expressed OMA1–HA, ∼40-kDa OMA1 was stabilised and very little ∼60 kDa OMA1 was detected after treatment with CCCP (Fig. 5B). By contrast, oligomycin caused the loss of ∼40 kDa OMA1 only in the OXPHOS-active RPE1 population (Fig. 5C) – which is consistent with the efficient cleavage of L-OPA1 in OXPHOS-dependent RPE1 cells that had been treated with oligomycin (Fig. 5C and Fig. 3C). We used protease protection assays to confirm that ∼60-kDa OMA1 resides on the OMM in RPE1 cells, from where it is susceptible to proteinase K proteolysis (Fig. 5D) (Head et al., 2009), and not at the IMM where L-OPA1 is located (McBride and Soubannier, 2010). Furthermore, we found that the levels of ∼40-kDa OMA1 were reduced in both glycolytic and OXPHOS-active cells upon the addition of cycloheximide (Fig. 5E), suggesting that there is a slow rate of basal OMA1 turnover that can be stimulated in unperturbed cells, where stimulation is dependent upon both the type of mitochondrial challenge and the cellular energy context. Crucially, the data from the cycloheximide and oligomycin experiments showed that efficient L-OPA1 processing is possible in the absence of detectable ∼60-kDa OMA1 (Fig. 5C,E), which provides an argument against ∼60-kDa OMA1 being the active protease. Hence, the degradation of ∼40-kDa OMA1, rather than accumulation of ∼60-kDa OMA1, correlates with energy-status-dependent processing of L-OPA1 following mitochondrial damage.

### OMA1-mediated cleavage of L-OPA1 is a crucial control point for mitophagy

**Fig. 6.**
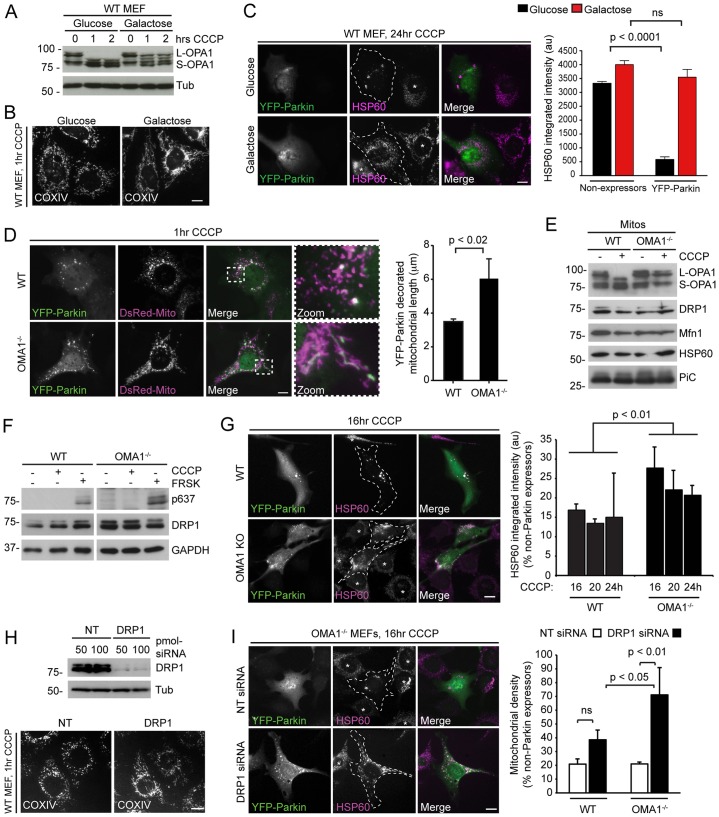
**Impaired Parkin-mediated mitophagy in OMA1^−/−^ MEFs.** (A) L-OPA1 is protected from inducible proteolytic processing in galactose-cultured MEFs that had been treated with 15 µM CCCP for 1–2 hours. Tub, tubulin. (B) COXIV immunostaining revealed that galactose-cultured MEFs preserve an elongated mitochondrial network after 1 hour of CCCP treatment. WT, wild type. (C) Mitophagy is blocked in galactose-cultured MEFs that had been transfected with YFP–Parkin. Cells were treated with CCCP for 24 hours and mitochondrial content was quantified in YFP–Parkin- and in non-expressing cells according to HSP60 integrated intensity measurements. Example images are shown on the left, quantification is shown on the right. (Means±s.d; *n* = 3; ≥50 cells; Student's *t*-test). (D) Maintenance of mitochondrial length in OMA1^−/−^ MEFs transiently expressing YFP–Parkin that had been treated with 15 µM CCCP. Example images are shown on the left, the insets show enlarged images of the indicated boxed area. Length measurements for YFP–Parkin-decorated mitochondria at 1 hour after treatment with CCCP are shown on the right (means±s.d.; *n* = 3; ≥125 cells; Student's *t*-test). (E) Mitochondria (Mitos) that had been isolated from wild-type (WT) and OMA1^−/−^ MEFs after treatment with or without CCCP (1 hour) were immunoblotted for the indicated proteins. Note the inefficient processing of L-OPA1 in the OMA1^−/−^ sample and the similar levels of mitochondrial DRP1 under all conditions. (F) Treatment with CCCP (1 hour) does not trigger phosphorylation of DRP1 at residue Ser637 (p637) in wild-type or OMA1^−/−^ MEFs (FRSK: forskolin positive control for PKA activation). (G) Mitophagy is delayed in OMA1^−/−^ MEFs. Example images at 16 hours CCCP treatment of wild-type and OMA1^−/−^ (OMA1 KO) MEFs that expressed YFP–Parkin (left panel), and quantification of mitochondrial content (HSP60 integrated intensity) that had been normalised against non-Parkin expressing cells (means±s.d.; *n* = 3, ≥60 cells; two-way ANOVA). au, arbitrary units. (H) DRP1 siRNA silencing in wild-type MEFs. NT, non-targeting. (I) Inhibition of mitophagy in OMA1^−/−^ MEFs in which DRP1 had been silenced. Images of OMA1^−/−^ MEFs that expressed YFP–Parkin (left panel), and quantification of mitochondrial density as a percentage of non-Parkin expressing cells are shown at 16 hours (means±s.d.; *n* = 3, ≥60 cells; Tukey's post-test). ns, not significant. Scale bars: 10 µm.

Our studies so far suggested that reduced OMA1-mediated cleavage of L-OPA1 at the IMM strongly reduces the efficiency of mitophagy in OXPHOS-dependent cells. Interestingly, L-OPA1 is constitutively cleaved in OXPHOS-active cells and tissues that lack the m-AAA protease AFG3L2 (Ehses et al., 2009; Maltecca et al., 2012). To test whether OMA1-dependent processing of L-OPA1 is both necessary and sufficient for stress-induced mitophagy, we, therefore, used siRNA to suppress AFG3L2 expression in OXPHOS-dependent RPE1 cells (Fig. 5F). Here, partial L-OPA1 processing was observed at steady state under these conditions (Fig. 5F), and this occurred alongside complete destabilisation of ∼40-kDa OMA1 in the absence of further stress (Fig. 5G), whereas the addition of CCCP stimulated complete L-OPA1 processing (Fig. 5F). Immunoblotting for the resident mitochondrial proteins COXIV and HSP60 was carried out as a measure of mitophagy. Silencing of AFG3L2 significantly reduced COXIV levels in galactose-cultured YFP–Parkin-expressing RPE1 cells that had been treated with CCCP for 20 hours (Fig. 5H), indicating that LOPA1 processing might be sufficient to facilitate mitophagy in cells that are dependent upon mitochondrial OXPHOS. By contrast, steady state HSP60 levels were markedly reduced in OXPHOS dependent RPE1 cells in which AFG3L2 had been silenced, but further reductions were not seen upon the addition of CCCP (Fig. 6I). This suggests that, similar to OMA1, HSP60 stability might depend on AFG3L2.

To further examine the functional requirements for L-OPA1 processing during mitophagy, we transiently expressed YFP–Parkin in wild-type MEFs and in OMA1^−/−^ MEFs, in which CCCP-induced L-OPA1 cleavage and mitochondrial fragmentation are markedly impaired (Quirós et al., 2012). Similar to RPE1 cells, wild-type MEFs that had been grown in galactose resisted complete L-OPA1 cleavage in response to CCCP (Fig. 6A), maintained a tubular mitochondrial network following mitochondrial depolarisation (Fig. 6B) and demonstrated inefficient YFP–Parkin-mediated mitophagy after 24 hours of treatment with CCCP (Fig. 6C). Importantly, YFP–Parkin was recruited to mitochondria with similar kinetics in wild-type and OMA1^−/−^ MEFs that had been treated with CCCP, although YFP–Parkin-positive mitochondria remained significantly longer in OMA1^−/−^ cells, which is indicative of impaired fission (Fig. 6D; supplementary material Movie 3). In addition, immunoblotting demonstrated that mitochondrial DRP1 levels were similar at steady state and after treatment with CCCP (Fig. 6E), indicating that the DRP1-mediated fission pathway is likely to be active in both cell types. Equally, residue Ser637 phosphorylation was not observed in wild-type or OMA1^−/−^ MEFs in the presence of CCCP (Fig. 6F). Hence, we conclude that the prevention of L-OPA1 cleavage is sufficient to protect against depolarisation-induced mitochondrial fragmentation in MEFs, perhaps, by restricting DRP1-mediated fission activity.

We next examined to what extent delaying mitochondrial fragmentation by blocking OMA1-dependent L-OPA1 processing influenced Parkin-mediated mitophagy. Crucially, mitophagy was significantly impaired in OMA1^−/−^ MEFs that transiently expressed YFP–Parkin up to 24 hours after CCCP addition (Fig. 6G). Because a combination of inefficient L-OPA1 processing and impaired DRP1 activity contribute to the inhibition of mitophagy in OXPHOS-dependent RPE1 cells, we asked whether blocking mitochondrial fission in the OMA1^−/−^ MEF background impairs the mitophagy pathway and effectively mirrors the scenario observed in OXPHOS-dependent cells. To test this, we suppressed DRP1 expression by using siRNA (Fig. 6H) and assessed mitophagy following 16 hours of treatment with CCCP in wild-type and OMA1^−/−^ MEFs cells that transiently expressed YFP–Parkin (Fig. 6I). In our hands, DRP1 suppression alone did not prevent mitophagy in wild-type MEFs, perhaps, due to incomplete DRP1 depletion, but it did significantly reduce the efficiency of mitophagy in OMA1^−/−^ MEFs (Fig. 6I). Importantly, DRP1-suppressed OMA1^−/−^ MEFs were significantly less efficient at executing mitophagy than DRP1-suppressed wild-type MEFs (Fig. 6I), further emphasising the importance of combined L-OPA1 cleavage and DRP1 fission for efficient stress-induced mitochondrial fragmentation and mitophagy (Fig. 6I).

## DISCUSSION

Here, we have explored the relationship between mitochondrial OXPHOS activity and mitophagy, and found that increased cellular dependency on OXPHOS causes a profound block of stress-induced mitophagy at the level of mitochondrial dynamics. For most of our studies, we used YFP–Parkin-expressing hTERT-immortalised RPE1 cells that clear their mitochondria with remarkable efficiency, making them a useful cell line in which to study the molecular and metabolic control of mitophagy. This efficiency might be explained by the major physiological role of the retinal pigment epithelium *in vivo*, which is to clear surrounding photoreceptor debris by using phagocytosis (Strauss, 2005) – a role in which autophagy has been implicated (Kim et al., 2013). Notably, cells of the retinal pigment epithelium are exposed to chronic oxidative stress as a consequence of exposure to light and high oxygen consumption (Shadrach et al., 2013). Protein and mitochondrial quality control might, therefore, be crucial evolutionary facets of cells that have been derived from the retinal pigment epithelium. In our RPE1-based model, the exchange of glucose for galactose corresponded with a shift away from glycolytic ATP production towards a metabolic dependency on OXPHOS. Under these conditions, RPE1 cells resisted Parkin-mediated mitophagy, despite the translocation of YFP–Parkin to uncoupled mitochondria, suggesting that the block in mitophagy occurs downstream of Parkin recruitment. This differs from the previous report of impaired mitochondrial Parkin recruitment in HeLa cells that had been grown in galactose and treated with CCCP (Van Laar et al., 2011) and suggests that different pathways exist to suppress mitophagy under OXPHOS conditions. While this manuscript was in revision, evidence for a block in PINK1- and Parkin-independent mitophagy in iron-depleted SH-SY5Y cells that had been grown in galactose was reported by Allen and colleagues, suggesting that the relationship between the dependency on OXPHOS and the control of mitophagy are widely observed (Allen et al., 2013). Interestingly, and unlike in RPE1 cells, autophagy and Nix-mediated mitophagy are dramatically upregulated in response to the switch to OXPHOS growth conditions in HeLa cells (Melser et al., 2013). One possible explanation is that changes in physiology and/or autophagy deregulation (White, 2012) during cellular transformation might result in an inefficient mitochondrial population that is prone to elevated levels of reactive oxygen species (ROS) generation in HeLa cells upon the stimulation of OXPHOS (Frank et al., 2012; Melser et al., 2013; Scherz-Shouval et al., 2007).

Previous research has highlighted that there is a requirement for mitochondrial fission in efficient mitophagy (Frank et al., 2012; Tanaka et al., 2010; Twig et al., 2008). In this regard, it is noteworthy that CCCP-induced fission was suppressed during OXPHOS-dependent growth in RPE1 cells. We identified parallel control pathways that are responsible for reduced fission – attenuated OMA1-dependent L-OPA1 cleavage and reduced DRP1 function. For the latter, our data implicate reduced steady state mitochondrial DRP1 levels and inhibitory PKA-mediated phosphorylation of residue Ser637 following dissipation of Δψ_m_, which would be similar to the regulatory pathways that have been described during mitochondrial hyperfusion in response to nutrient starvation (Gomes et al., 2011; Rambold et al., 2011). The signalling pathways that act upstream of PKA in the context of mitochondrial stress remain obscure. PKA is stimulated by elevated levels of intracellular cAMP, which is, classically, derived from glucagon-mediated activation of G-protein-responsive transmembrane-spanning adenylyl cyclases at the plasma membrane (Unger, 1985); however, the emergence of soluble and intra-mitochondrial adenylyl cyclases mean that there could be a number of alternative pathways for elevated cAMP levels in response to the damage to active mitochondria (Acin-Perez et al., 2009). Of note, increased PKA activity has been linked to mitochondrial dysfunction in yeast (Graef and Nunnari, 2011). Phosphorylation of DRP1 at residue Ser637 can result from suppressed calcineurin phosphatase activity or from a post-translational modification that prevents calcineurin from binding to the LxVP motif on DRP1 (Slupe et al., 2013). Calcineurin inhibition upon loss of Δψ_m_ would be unexpected given that treatment with CCCP has been shown to increase intracellular Ca^2+^ and stimulate calcineurin activation *in vitro* (Cereghetti et al., 2008). Recently, the overexpression of the fission factors MiD49 and MiD51 was shown to sequester inactive YFP–DRP1 puncta on mitochondria in a dominant-negative fashion (Palmer et al., 2013), demonstrating that the creation of YFP–DRP1 foci does not always indicate future sites of fission. MiD49 and MiD51 overexpression has also been shown to result in phosphorylation of DRP1 at Ser637 (Losón et al., 2013); therefore, the exploration of MiD49 and MiD51 activity in OXPHOS-active cells would further elucidate the mechanism of this process.

Under conditions of OXPHOS, RPE1 cells did not activate OMA1 upon treatment with CCCP but did activate efficient proteolysis of L-OPA1 after treatment with oligomycin or depletion of AFG3L2. Our analysis of OMA1 processing and mitochondrial distribution supports a model in which ∼40-kDa OMA1 is the active protease and ∼60-kDa OMA1 is generated at the OMM due to attenuated mitochondrial import. L-OPA1 cleavage is closely linked to the abrupt degradation of ∼40-kDa OMA1, suggesting activation of an upstream protease and/or OMA1 auto-proteolysis. By contrast, in OXPHOS-active RPE1 cells in which AFG3L2 had been suppressed, ∼40-kDa OMA1 was efficiently degraded in the absence of L-OPA1 cleavage. This sudden turnover of ∼40-kDa OMA1 is necessary but not sufficient for L-OPA1 processing, suggesting that the access of active OMA1 to L-OPA1 is tightly regulated; a scenario that fits well with the proposed model of IMM sub-domains that are established by the actions of prohibitin family proteins that constrain protein lateral mobility in the plane of the membrane (McBride and Soubannier, 2010). AFG3L2 might, therefore, influence OMA1–L-OPA1 interactions at the IMM. One possible mechanism for this is through the regulation of the hypoxia-induced gene domain protein-1a (Higd-1a), which has been shown to contribute to OPA1 stability (An et al., 2013). Overexpression of Higd-1a in cultured cells delayed L-OPA1 cleavage in response to CCCP, whereas its suppression enhanced L-OPA1 proteolysis (An et al., 2013). Crucially, the effect of Higd-1a on OMA1 regulation has not been investigated, but its direct interaction with OPA1 might be a key mechanism for regulating OPA1 processing in response to mitochondrial stress. Interestingly, OMA1 proteolytic control appears to be an intrinsic property in mammals, because this is not observed in yeast (Khalimonchuk et al., 2012). Nevertheless, OMA1 activity might still be directly regulated by changes in Δψ_m_ in both mammals and yeast.

Here, our studies of OMA1^−/−^ MEFs, which are incapable of cleaving L-OPA1 in response to mitochondrial stress (Quirós et al., 2012), reveal a substantial delay in mitochondrial fragmentation at early timepoints after treatment with CCCP, despite the presence of similar levels of mitochondrial DRP1. Live-cell imaging suggests that this was due to a delay in fission, rather than an upregulation of fusion. Although this will require further confirmation, a requirement for L-OPA1 cleavage at the IMM, upstream of DRP1 activity, would be extremely important. In this regard, it is interesting that in OXPHOS-dependent RPE1 cells that stably expressed YFP–DRP1, we observed the generation of numerous, apparently ineffectual, DRP1 puncta in the vicinity of mitochondrial laser photodamage. Additional studies will be required to test this idea, not least, because we could not confirm whether L-OPA1 was processed locally to laser photodamage in the glucose-cultured control cells. Notably, silencing of DRP1 expression in OMA1^−/−^ MEFs revealed that the combined inactivation of DRP1 and OMA1 restricted Parkin-mediated mitophagy to a much greater extent than suppression of DRP1 alone. These data highlight the physiological importance of the bioenergetic control of OMA1 and DRP1 activity, revealed in glucose- and galactose-cultured RPE1 cells.

## MATERIALS AND METHODS

### Cell lines, tissue culture and reagents

Cell lines were maintained at 37°C and under 5% CO_2_. RPE1 cells were cultured in Dulbecco's modified Eagle's Medium (DMEM) (11966-025, Gibco) that had been supplemented with 10% foetal bovine serum (FBS), 0.5 mM sodium pyruvate, 5 mM HEPES, 1% penicillin and streptomycin, and either 10 mM glucose or 10 mM galactose. Cells were passaged in glucose or galactose at least three times before experiments. Mouse embryonic fibroblasts (MEFs) were cultured in DMEM (D5796, Sigma) and 10% FBS. The following chemicals were used: BafA1 (Enzo Life Sciences); carbonyl cyanide *m*-chlorophenyl hydrazone (used at 10 µM unless otherwise stated, Sigma-Aldrich), cycloheximide (CHX, 1 µg/ml, Sigma); forskolin (20 µM, Enzo Life Sciences); oligomycin (Sigma); and H89 (10 µM, Tocris Biosciences).

### Constructs and transfection

YFP–Parkin was a kind gift from Richard Youle (National Institute of Neurological Disorders and Stroke, MD). Mito–CFP was generated using a 41-amino-acid mitochondrial-targeting motif of human Atg4D (Betin et al., 2012) cloned into pECFP-N1. The mCherry-GFP-LC3 construct was made by inserting GFP between mCherry and LC3b (Map1lc3xx) in a pmCherry-C1-LC3 background. YFP–DRP1 was a kind gift from Luca Scorrano (Department of Biology and Dulbecco-Telethon Institute, Venetian Institute of Molecular Medicine, University of Padova, Italy, Geneva) and pSPORT6 OMA1-HA was kindly provided by Alex van der Bliek (University of California, Los Angeles, CA). For lentiviral production and stable cell line generation, constructs were cloned into the pLVX-puro plasmid and lentivirus was harvested from HEK293T cells using the Lenti-X HTX lentiviral expression system (Clontech) according to the manufacturer's instructions. Target cells were infected with lentivirus and treated with 1–10 µg/ml puromycin for selection. MEFs were transiently transfected with DNA and siRNA using the Neon Transfection System (pulse voltage, 1350 V; pulse width, 30 ms; pulse cycles, 1; DNA, 1–3 µg; siRNA, 50 pmol; Life Technologies). siRNA reverse-transfection in RPE1 cells was performed using Lipofectamine 2000 (Life Technologies). siGENOME SMARTpools (Thermo Scientific) of siRNA against AFG3L2 (M-005781-00-0005) and non-targeting siRNA (D-001206-14-05) were used. DRP1 knockdown experiments were performed using siRNA against DRP1 (GCAGAAGAAUGGGGUAAAU) and a negative control siRNA targeting luciferase (CGUACGCGGAAUACUUCGA).

### Antibodies

The primary antibodies used were: mouse monoclonal against COXIV subunit I (ab14705, Abcam), mouse monoclonal against HSP60 (H4149, Sigma), rabbit polyclonal against LAMP1 (a gift from Ash Toye, University of Bristol, Bristol, UK); rabbit polyclonal against LC3 (642157, Viva Bioscience), mouse monoclonal against SQSTM1/p62 (H00008878-M01, Abnova), mouse monoclonal against Tom20 (612278, BD Biosciences); mouse polyclonal against AFG3L2 (ab68023, Abcam), mouse monoclonal against DRP1 (611112, BD Biosciences), rabbit polyclonal against DRP1 phosphorylated Ser637 (4867, Cell Signaling), mouse monoclonal against GAPDH (G8795, Sigma), rabbit polyclonal against HA (600-401-384, Rockland), rabbit polyclonal against PiC (a gift from Andrew Halestrap, University of Bristol, Bristol, UK), mouse monoclonal against Mfn1 (ab57602, Abcam), mouse monoclonal against OPA1 (612607, BD Biosciences), mouse monoclonal against tubulin (T5168; Sigma), mouse monoclonal against WIPI2 (MCA5780GA, AbD Serotec). The secondary antibodies used were: goat against mouse IgG conjugated to Alexa Fluor 594 (A-11032, Molecular Probes, Life Technologies), goat against rabbit IgG conjugated to Alexa Fluor 594 (A-11037, Molecular Probes, Life Technologies); goat against mouse IgG conjugated to Alexa Fluor 488 (A-11029, Molecular Probes, Life Technologies); donkey against mouse IgG conjugated to horseradish peroxidase (HRP) (715-035-150, Jackson Immunoresearch Laboratories), donkey against rabbit IgG conjugated to HRP (711-035-152, Jackson Immunoresearch Laboratories).

### Imaging

Wide-field images were obtained by using an Olympus IX-71 inverted microscope (60× Uplan Fluorite objective, 1.25 NA, oil) fitted with a CoolSNAP HQ CCD camera (Photometrics) driven by MetaMorph software (Molecular Devices). For live-cell imaging, cells were seeded on 35-mm glass-bottomed dishes (MatTek Corp) at least one day before imaging. LC3- and WIPI2-labelled puncta were counted from wide-field images using MetaMorph software. Raw images were processed using the TopHat morphology filter, selecting circular profiles of less than 5 pixels (Betin and Lane, 2009). For mitochondrial staining, cells were incubated in medium containing either 50 nM MitoTracker CMX-ROS (Life Technologies) or 50 ng/ml tetramethylrhodamine, ethyl ester (TMRE) for 15 minutes. Wide-field live-cell imaging was carried out at 37°C under 5% CO_2_. Time-lapse confocal images of YFP–DRP1-expressing RPE1 cells that had been loaded with MitoTracker were acquired using a PerkinElmer UltraVIEW ERS 6FE spinning disc confocal system attached to a Leica DM I6000 inverted epifluorescence microscope (100× objective, 1.4 NA, oil). A Hamamatsu C9100-50 EM-CCD camera was used and the microscope was driven by Volocity software (Improvision). Laser photodamage was induced by using the PerkinElmer photokinesis accessory with a 488-nm 14-mW argon laser on standby at 100% power. Laser scans were targeted to a 15-pixel^2^ ellipse, set for the duration of 1 ms and cycled 50 times. One pre-bleach image was acquired and post-bleach images of YFP–DRP1 and MitoTracker were captured at one focal plane every 2 seconds for 2 minutes.

### Mitochondrial isolation and parameters

Mitochondria were isolated by differential centrifugation as previously described (Betin et al., 2012). To measure mitochondrial length, images of MitoTracker-CMX-ROS- or Tom20-labelled mitochondria were obtained by wide-field microscopy and analysed by using Metamorph software, using a method adapted from Song and colleagues (Song et al., 2008). Briefly, out of focus light was removed using Fourier transformation and this was followed by binarisation, thresholding and skeletonisation. This resulted in mitochondria being displayed in binary as 1-pixel-thick shapes (single pixels were discarded from the skeletonised images). Each mitochondrion was defined as a single region of interest by the software and length measurements were calculated. To measure mitochondrial density in OMA1**^−/−^** MEF experiments, images of MEFs labelled with HSP60 were acquired by wide-field microscopy using consistent exposure settings (2×binning) and analysed using Metamorph software. Non-transfected cells and YFP–Parkin-expressing cells were distinguished and thresholded according to HSP60 staining intensity. Binary images were generated, and the integrated intensity calculated for each cell.

### Measurement of OXPHOS and glycolysis

All measurements were performed with a Seahorse Bioscience XF24 Extracellular Flux Analyzer at the Wolfson Institute for Biomedical Research (University College London, London, UK). This system allowed simultaneous measurement of cellular oxygen consumption rate (OCR in pmoles/minute) and extracellular acidification rate (ECAR in mpH/minute). Cells were plated at 40,000 cells/well onto Seahorse 24-well plates 48 hours before the assay. Initial assays were used to optimise cell number, CCCP concentration, oligomycin concentration and mix-wait-measure cycle durations (not shown). The typical mitochondrial stress test was performed as follows: the baseline OCR and ECAR was measured at three timepoints in at least five replicate wells for each condition. Oligomycin (0.75 µM) was then injected to block ATP-synthase, resulting in inhibited oxygen consumption and electron flow. CCCP (0.5 µM) was next injected to uncouple electron transport from ATP synthesis by shuttling protons across the inner mitochondrial membrane. Finally, the complex I inhibitor rotenone (1 µM) was injected to block electron transport and terminate oxygen consumption.

### ATP measurements

Cells were harvested by scraping into ice-cold PBS and then pelleted by centrifuging at 1000 r.p.m. for 5 minutes. Cells were then lysed by resuspending in urea and CHAPs lysis buffer [9M urea, 2% CHAPS in 30 mM Tris with 1× protease inhibitor cocktail (Roche)]. Samples were vortexed and briefly spun at 20,000 g. The ATP levels in fresh samples were determined using an ATP determination kit (Invitrogen) according to the manufacturer's instructions. Luminescence was recorded using an L Max II Luminometer (Molecular Devices) in a 96-well format. ATP concentration was normalised to the protein concentration that was measured by the Lowry-based detergent compatible (DC) protein assay (BioRad).

## Supplementary Material

Supplementary Material
