# Supplementary material for: Impaired OMA1-dependent cleavage of OPA1 and reduced DRP1 fission activity combine to prevent mitophagy in cells that are dependent on oxidative phosphorylation
Source: J Cell Sci. 2014 May 15;127(10):2313–25. doi: 10.1242/jcs.144337 (PMC4021475; doi:10.1242/jcs.144337)
Supplement: Supplementary Material [file supp_127_10_2313__index.html]

Impaired OMA1-dependent cleavage of OPA1 and reduced DRP1 fission activity combine to prevent mitophagy in cells that are dependent on oxidative phosphorylation — Supplementary Material 

# Impaired OMA1-dependent cleavage of OPA1 and reduced DRP1 fission activity combine to prevent mitophagy in cells that are dependent on oxidative phosphorylation

## JCS144337 Supplementary Material

**Files in this Data Supplement:**

- **Supplementary Material**
