# Supplementary material for: Impaired OMA1-dependent cleavage of OPA1 and reduced DRP1 fission activity combine to prevent mitophagy in cells that are dependent on oxidative phosphorylation
Source: J Cell Sci. 2014 May 15;127(10):2313–25. doi: 10.1242/jcs.144337 (PMC4021475; doi:10.1242/jcs.144337)
Supplement: Supplementary Material [file supp_127.10.2313_JCS144337.pdf]

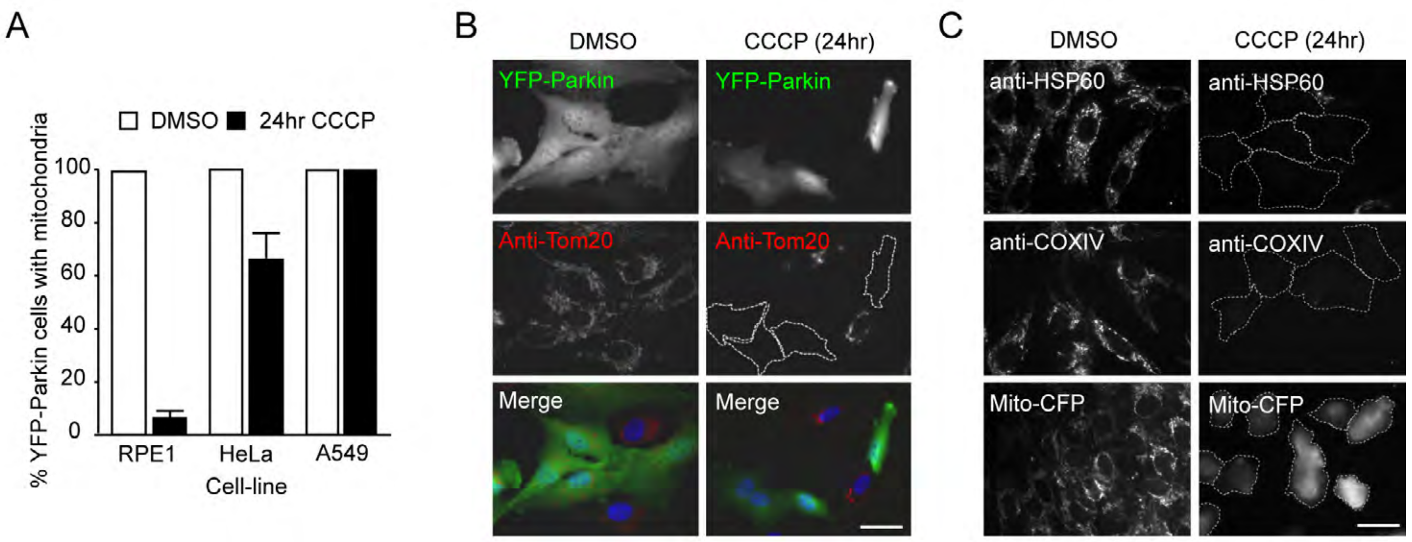


**Fig. S1. Parkin-mediated mitophagy in RPE1 cells.** (A) Mitochondrial clearance analysis in 3 representative human cell-lines stably expressing YFP-Parkin. After 24 hours treatment with DMSO (vehicle control) or CCCP, cells were fixed and stained with antibodies against Tom20. Wide-field fluorescence imaging was used to score numbers of cells with mitochondria (*n*=3). (B, C) Wide-field fluorescence imaging of mitochondria in RPE1 cells expressing YFP-Parkin treated with DMSO or CCCP for 24 hours. Bars=20 ∀m.

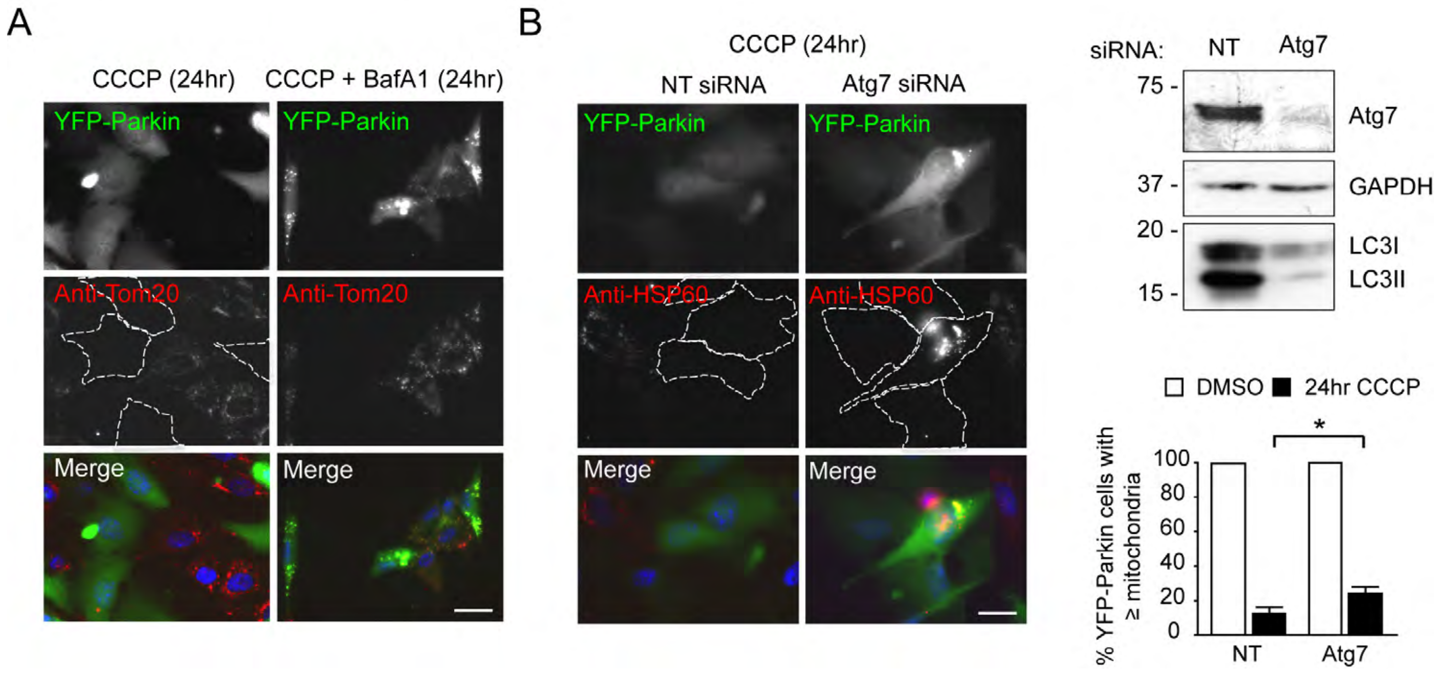


**Fig. S2. Blocking the autophagy pathway prevents mitophagy in YFP-Parkin RPE1 cells.** (A) Parkin-mediated mitophagy is blocked by the lysosomal vacuolar ATPase inhibitor, BafA1. (B) Silencing the essential autophagy gene, Atg7, partially blocks Parkin-mediated mitophagy in RPE1 cells. Example images to the left; immunoblot of Atg7 siRNA suppression and mitophagy quantitation to the right. Bars=20 ∀m.

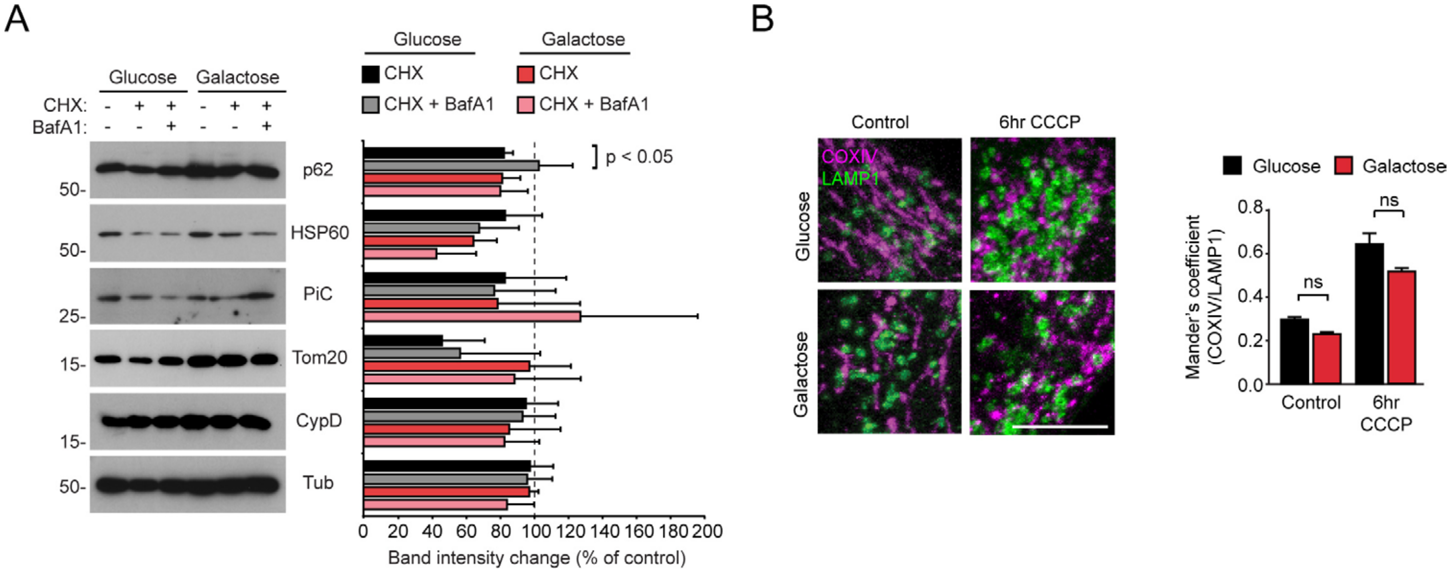


**Fig. S3. Basal mitophagy rates are not altered in OXPHOS-dependent RPE1 cells.** (A) Immunoblot analysis of mitochondrial protein levels in wild type RPE1 cells cultured in glucose or galactose media. Cells were incubated with CHX in the absence or presence of BafA1 (8 hours) (*n*=3; means ± s.d.; student's *t*-test). (B) Colocalisation analysis of mitochondria and lysosomes in glucose and galactose cultured wild type RPE1 cells, in the absence or presence of CCCP (Mander's coefficient; *n*=30 cells; student's *t*-test). Bars=10 ∀m.

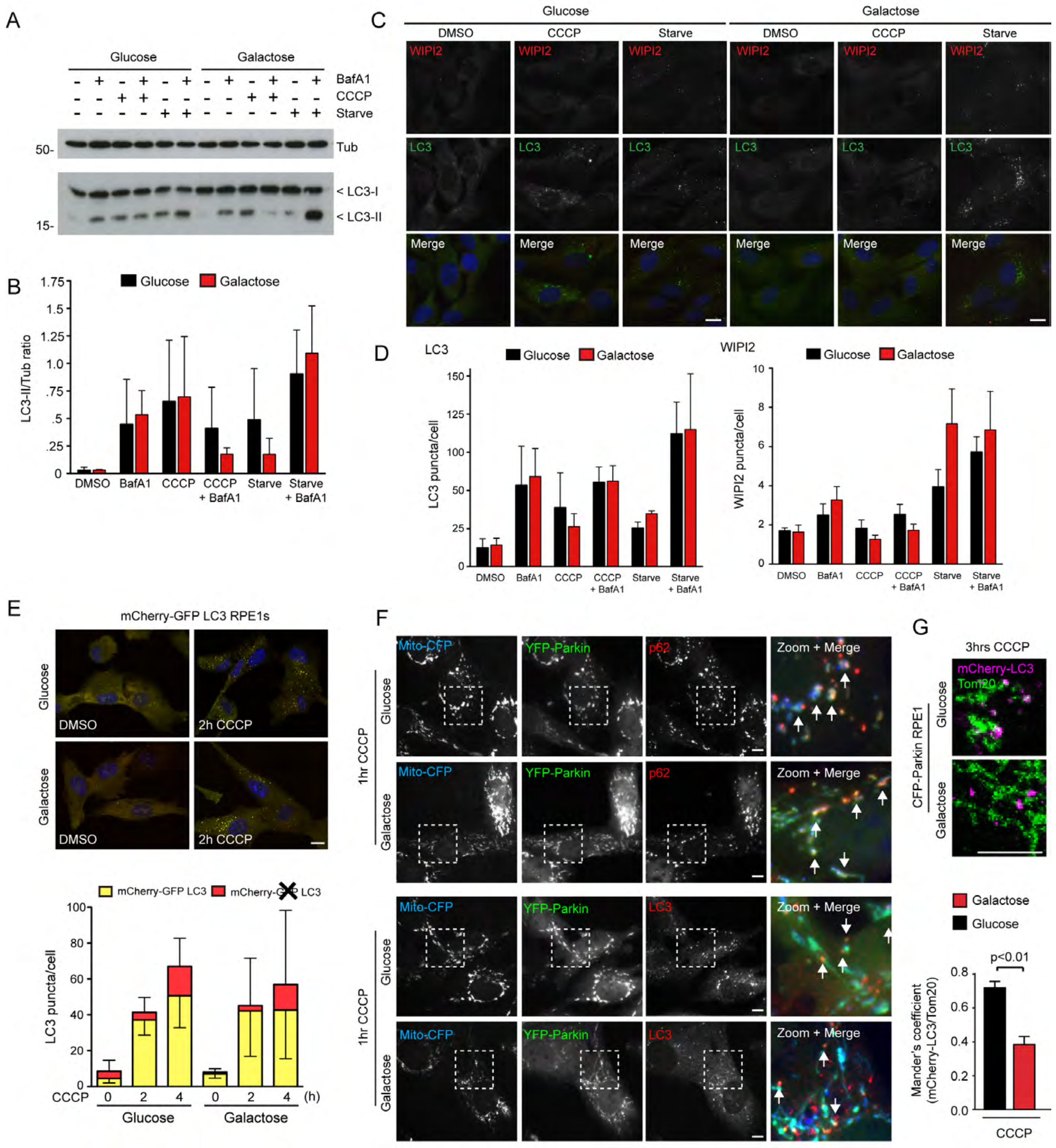


**Fig. S4. A functional autophagy pathway in OXPHOS-dependent RPE1 cells.** (A, B) Autophagy responses in glucose and galactose cultured RPE1 cells measured by immunoblotting for lipidated LC3-II. Cells were treated with 10 ∀M CCCP or amino acid starved in the absence or presence of BafA1 for 1.5 hours. (B) Quantification of the ratio of LC3-II/Tub revealed similar autophagy responses between glucose and galactose cultured RPE1 cells (*n*=3). (C, D) Autophagy responses in glucose and galactose cultured RPE1 cells measured by simultaneous immunostaining for LC3 and WIPI2. Cells were treated with 10 ∀M CCCP or amino acid starved in the absence or presence of BafA1 for 1.5 hours. (C) Example widefield images. (D) Quantification of LC3 and WIPI2 puncta numbers revealed that glucose and galactose cultured RPE1 cells respond similarly to autophagy induction (*n*=3; ≥280 cells). (E) Autophagic flux is not altered in galactose-cultured RPE1 cells stably expressing mCherry-GFP-LC3 (~280 cells analysed across 2 experiments ±s.d.). (F) Evidence for p62 and LC3 recruitment to Parkin decorated mitochondria in CCCP-treated RPE1 cells stably expressing Mito-CFP and YFP-Parkin. Cells were fixed and stained with antibodies against p62 or LC3. Arrows indicate areas of colocalisation. (G) Colocalisation analysis of mitochondrial (anti-Tom20) and autophagosomal (mCherry LC3) markers following CCCP treatment in glycolytic and OXPHOS-dependent RPE1 cells stably expressing CFP-Parkin (for the sake of clarity, the CFP-Parkin image is not shown). Cells were treated with CCCP for 3 hrs. (*n*=3; ≥70 cells; means ± s.d.; one-way ANOVA, Tukey's post test). Bars=10 ∀m.

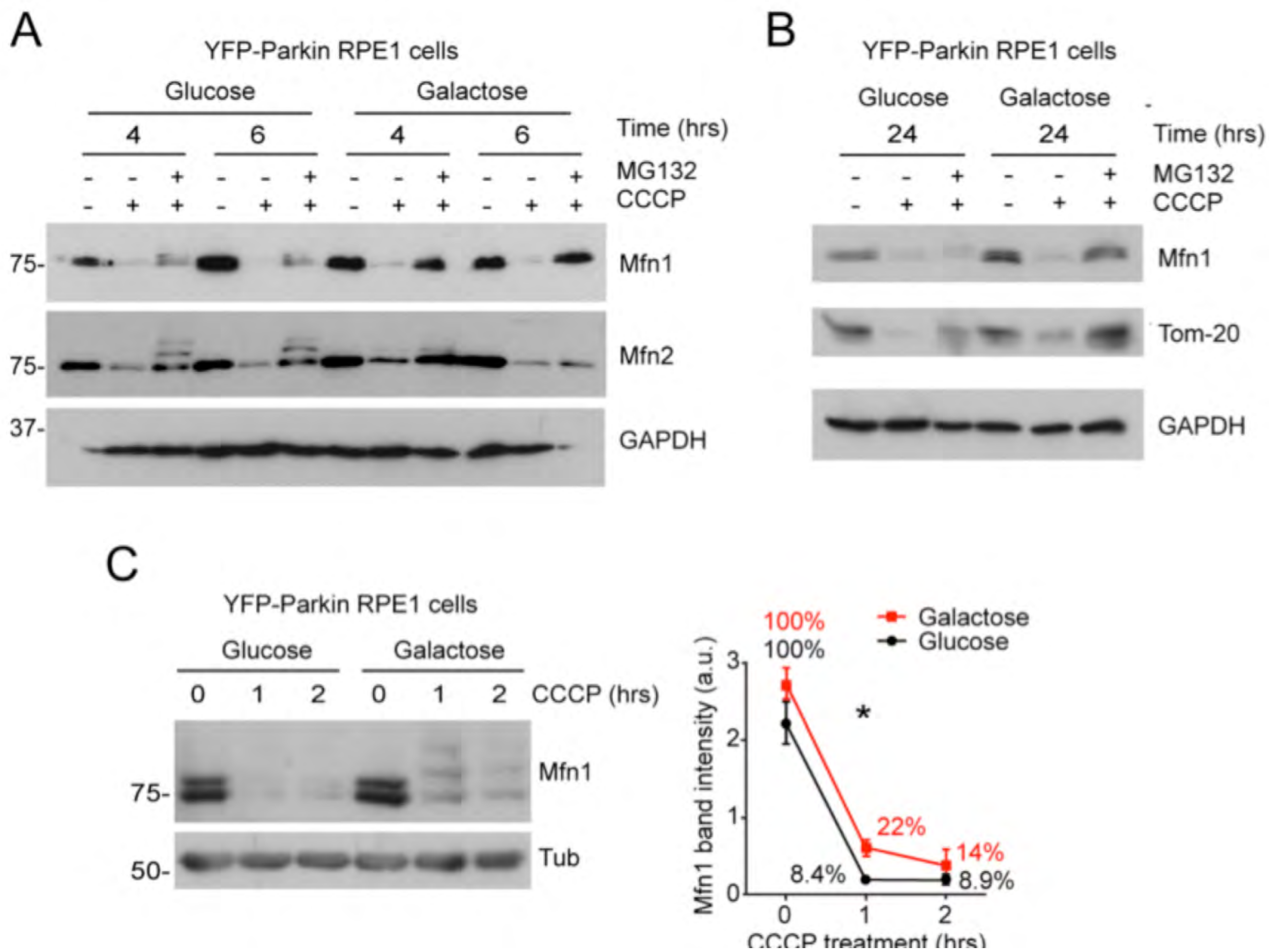


**Fig. S5. Parkin-mediated mitofusin degradation in glucose and galactose.** (A, B) Evidence for proteasome-mediated mitofusin degradation under both glycolytic and OXPHOS-dependent conditions. Cells were treated for the times shown with CCCP in the absence or presence of the proteasome inhibitor MG132. The loss of Tom20 in the glucose cultured population at 24 hrs in the presence of MG132 is probably due to mitophagy. (C) Kinetics of Mfn1 degradation in glucose and galactose. Example immunoblot to the left; densitometry quantitation to the right (*n*=3) (2-way ANOVA, Bonferroni post test).

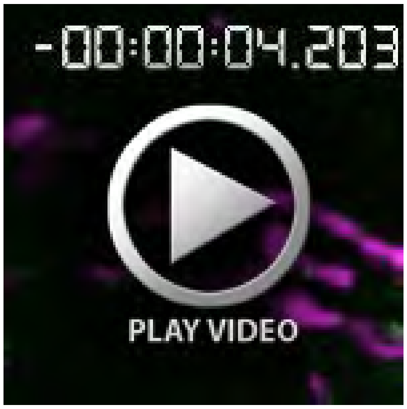


**Movie 1. YFP-DRP1 recruitment and fission dynamics in a glucose-cultured RPE1 cells.** A selected mitochondrion in an RPE1 cells stably expressing YFP-Parkin and loaded with MitoTracker, laser photodamaged and subsequently imaged by spinning disc confocal microscopy (Perkin Elmer UltraVIEW ERS 6FE; Hamamatsu C9100-50 EM-CCD camera; frames taken at 1.6 sec intervals for 2 min). Movie is linked to Fig. 4D. Bar=5 ∀m.

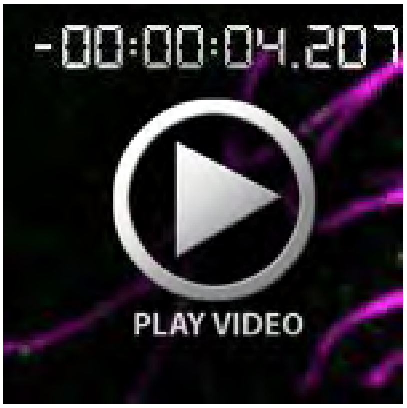


**Movie 2. YFP-DRP1 recruitment and fission dynamics in a galactose-cultured RPE1 cells.** A selected mitochondrion in an RPE1 cells stably expressing YFP-Parkin and loaded with MitoTracker, laser photodamaged and subsequently imaged by confocal microscopy (Perkin Elmer UltraVIEW ERS 6FE; Hamamatsu C9100-50 EM-CCD camera; frames taken at 1.6 sec intervals for 2 min). Movie is linked to Fig. 4D. Bar=5 ∀m.

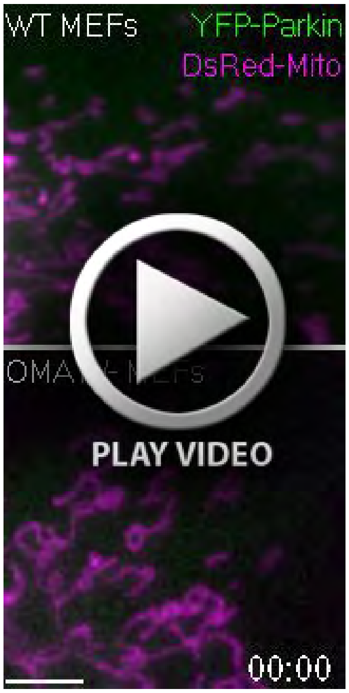


**Movie 3. Mitochondrial fragmentation and YFP-Parkin recruitment in wild type and OMA1$^{-/-}$ MEFs treated with CCCP.** Wild type and Oma1$^{-/-}$ MEFs were transiently transfected with YFP-Parkin and DsRed-Mito. Cells were treated with 15 ∀M CCCP at t=0 (Olympus IX-71 wide-field microscope; Photometrics CoolSNAP HQ2 CCD camera; frames taken at 2 min intervals for 2 h). Bar=10 ∀m.
